# Unequal exposure: spatio-temporal and structural determinants of personal PM_2.5_ exposure in three sub-Saharan African geographies—the Gambia, Kenya, and Mozambique

**DOI:** 10.3389/fgwh.2026.1843221

**Published:** 2026-07-30

**Authors:** Liberty Makacha, Yiqun Han, P. Tatenda Makanga, Cathryn Tonne, Marie-Laure Volvert, Hawanatu Jah, Yahaya Idris, Esperança Sevene, Moses Mukhanya, Angela Koech, Onesmus Wanje, Anifa Valá, Hannah Blencowe, Umberto d’Alessandro, Marleen Temmerman, Anna Roca, Jeffrey N. Bone, Laura A. Magee, Peter von Dadelszen, Ben Barratt, the PRECISE and PRECISE-DYAD Networks

**Affiliations:** 1 Department of Women and Children's Health, King's College London, London, United Kingdom; 2 Place Alert Labs, Midlands State University, Gweru, Zimbabwe; 3 School of Public Health, Imperial College London, London, United Kingdom; 4 Centre for Sexual Health and HIV/AIDS Research Zimbabwe (CeSHHAR), Harare, Zimbabwe; 5 Department of International Public Health, Liverpool School of Tropical Medicine, Liverpool, United Kingdom; 6 ISGlobal, Barcelona Institute for Global Health, Universitat de Barcelona, Barcelona, Spain; 7 MRC Unit The Gambia at the London School of Hygiene and Tropical Medicine, Banjul, The Gambia; 8 Centro de Investigação em Saúde de Manhiça, Manhiça, Mozambique; 9 Faculty of Medicine, Eduardo Mondlane University, Maputo, Mozambique; 10 Centre of Excellence for Women and Child Health, East Africa, Aga Khan University, Nairobi, Kenya; 11 London School of Hygiene and Tropical Medicine, London, United Kingdom; 12 ICREA, Barcelona, Spain; 13 British Columbia Children's Hospital Research Institute, Vancouver, BC, Canada

**Keywords:** air pollution, personal exposure, PM₂.₅, pregnancy, sub-Saharan Africa, wearable monitoring

## Abstract

**Background:**

Personal exposure to fine particulate matter (PM_2.5_) in sub-Saharan Africa remains poorly characterised, obscuring the roles of structural inequities, seasonality, and mobility in shaping dose.

**Methods:**

We undertook 5day per season personal PM_2.5_ monitoring among 343 parous women in The Gambia, Kenya, and Mozambique, spanning dry and wet seasons. Minute-resolved personal concentrations were aligned with microenvironment, fuel use, occupation, wealth, and meteorology within an integrated exposure assessment framework incorporating microenvironment classification and structural determinants; ambient PM_2.5_ was measured at nearby health facilities.

**Results:**

Across 3,190 person-days, daily median PM_2.5_ was 10.4 (7.6–14.7) μg/m^3^ in The Gambia, 8.1 (6.8–9.8) μg/m^3^ in Kenya, and 10.9 (9.3–13.5) μg/m^3^ in Mozambique. Episodic spikes were prominent. WHO 24-h health guideline exceedances were strongly seasonal and site-specific (The Gambia: 55.9% dry vs. 13.6% wet; Kenya: 8.9% vs. 3.7%; Mozambique: 20.9% vs. 28.6%). Exposures were higher away from home, with mid-day peaks (≈12:00–16:00). At home, biomass/coal users had higher medians than LPG/electricity users. Structural gradients were evident, including population density, occupation, and wealth gradients. Ambient–personal concordance was heterogeneous and context-dependent (*R*^2^ 0.01–0.56; slopes <1).

**Conclusion:**

Personal PM_2.5_ exposure among pregnant women in these three African settings was shaped by season, mobility, household energy, and structural disadvantage. Household clean-fuel transitions remain essential but are unlikely to fully reduce exposure unless complemented by interventions targeting transport corridors, markets, peri-domestic combustion, and other away-from-home exposure environments.

## Introduction

### Climate-structured air pollution exposure pathways in maternal health research in sub-Saharan Africa

Climate variability in Sub-Saharan Africa—manifested through seasonal shifts in precipitation, wind dynamics, temperature, and dust mobilisation—fundamentally shapes air pollution profiles. In many settings, these climatic drivers intersect with structural determinants such as biomass fuel reliance, informal labour, and rapid urbanisation to generate highly heterogeneous exposure environments for pregnant women. Yet maternal exposure assessment in the region remains dominated by sparse ambient monitoring and modelled estimates, limiting understanding of how climatic conditions translate into personal dose across microenvironments and social strata.

Critical gaps persist in three areas: (i) high-resolution personal exposure measurement in African maternal populations; (ii) quantification of socioeconomic and rural–urban patterning of exposure within shared climatic contexts; and (iii) characterisation of episodic high-intensity exposures that may be relevant for acute cardiovascular and placental stress. Without such evidence, climate-responsive maternal health strategies risk being insufficiently targeted or seasonally misaligned.

This study addresses these gaps by characterising minute-resolution personal PM_2.5_ exposure across three climatically and socio-structurally distinct Sub-Saharan African contexts. This represents one of the most comprehensive multi-country personal PM_2.5_ datasets currently available in Sub-Saharan Africa. By integrating seasonal contrasts, microenvironmental classification, inequality metrics, and ambient–personal concordance analyses, the work advances exposure science in support of climate-informed maternal and newborn health policy. It reveals how structural vulnerability and microenvironmental factors shape exposure and quantifies the limitations of ambient proxies.

The findings provide a foundation for seasonally adaptive surveillance, structurally targeted mitigation, and strengthened monitoring systems in under-monitored African settings. Personal air pollution exposure in sub-Saharan Africa must be addressed through seasonally targeted, context-specific interventions that go beyond household fuels. Policymakers must prioritise informal work environments, seasonal emissions, and housing infrastructure in interventions that seek to reduce exposure vulnerabilities.

### Limitations of ambient-centric exposure models in sub-Saharan Africa

Despite growing recognition of air pollution as a major determinant of maternal and perinatal health, exposure assessment in sub-Saharan Africa remains constrained by reliance on ambient monitoring networks and modelled estimates. These approaches inadequately capture the spatio-temporal variability and behavioural determinants that shape individual-level exposure, particularly in settings characterised by high mobility, mixed fuel use, and complex microenvironmental conditions (1).

Fine particulate matter (PM_2.5_ is of particular concern due to its deep pulmonary penetration and wide-ranging impacts on cardiovascular, respiratory, and perinatal health (2). Despite the growing body of evidence linking PM_2.5_ exposure to adverse health outcomes, global exposure science remains overwhelmingly skewed towards high-income settings (3). Sub-Saharan Africa—home to over 1.1 billion people and some of the fastest growing urban populations in the world—remains a critical blind spot (4, 5). In sub-Saharan Africa, household reliance on biomass fuels, poorly ventilated housing, rapid urbanisation, informal economies, and weak regulatory frameworks contribute to complex and context-specific exposure profiles (6). Conventional exposure assessment approaches, which rely heavily on ambient monitoring networks and satellite-derived estimates, often fail to capture the reality of lived exposure in these settings—where indoor sources, mobility patterns, and micro-environmental dynamics dominate (7, 8). Therefore, the burden of air pollution is underestimated and mischaracterised in ways that limit the effectiveness and equity of interventions (9).

Few studies have employed personal exposure monitoring in sub-Saharan Africa, and even fewer have done so across multiple countries, seasons, and micro-environmental contexts (10). This lack of high-resolution, person-centred data has hindered both the development of accurate exposure models and the design of culturally and structurally relevant interventions. There is limited understanding of how structural determinants—such as socio-economic status, occupation, energy access, and rural–urban residence—interact with environmental variables to shape disparities in personal PM_2.5_ exposure (11).

This manuscript presents the results of comprehensive field campaigns in The Gambia, Kenya, and Mozambique, aimed at capturing both personal and ambient air pollution measurements in seven peri-urban and rural settings. The study was nested within the PRECISE birth and women's health cohort study, providing access to vulnerable populations and their communities (12, 13).

The objectives of this study were fourfold:

1. to quantify cross-country and seasonal variability in personal PM_2.5_ exposure using high-resolution monitoring;
2. to characterise structural patterning of exposure across wealth status, occupation, and rural–urban residence;
3. to partition exposure by microenvironment using geolocation-informed classification; and
4. to evaluate concordance between personal and ambient PM_2.5_ concentrations in sites with reference monitoring.

By integrating climatic variability, structural determinants, and microenvironmental exposure pathways, this study aims to advance climate-informed exposure science and support more equitable maternal health policy in Sub-Saharan Africa. Positioned within exposure measurement and analytical methods research, this study advances a high-resolution, context-aware exposure assessment framework for maternal populations in Sub-Saharan Africa. By integrating wearable sensor data, geolocation-informed microenvironment classification, and structural determinants, we characterise complex interactions between environmental, behavioural, and socio-economic drivers of exposure. This approach addresses key limitations of conventional exposure models by incorporating precise temporal resolution, mobility-linked exposure pathways, and within-population heterogeneity, thereby supporting improved epidemiological inference and intervention targeting in maternal and newborn health research.

## Methods

### Study design and setting

This multi-country prospective observational study with integrated environmental exposure assessment was implemented as a sub-component of the PRECISE (PREgnancy Care Integrating translational Science, Everywhere) pregnancy cohort (12, 14). Participants were recruited from three Sub-Saharan African countries—The Gambia, Kenya, and Mozambique—purposefully selected to capture heterogeneity in geography, climatic regimes, settlement structure, infrastructure, and household energy access. Field protocols were adapted to reflect contextual realities of exposure measurement in low-resource settings.

### Participant selection and monitoring

Eligible participants were parous women from the PRECISE cohort who consented to participate in the air quality sub-study. Each participant underwent two rounds of personal exposure monitoring, timed to represent opposing seasons (locally defined dry and wet seasons). Each monitoring round was designed to capture approximately five days of personal exposure data per participant per season. These measurements therefore represent repeated short-term seasonal exposure profiles rather than continuous exposure assessment across the entire pregnancy. Stratified sampling ensured representation across household fuel types, occupational roles, and socio-economic strata.

Personal exposure to PM_2.5_ was measured using high-resolution, Dyson wearable monitors equipped with Sensirion SPS30 laser optical sensors (Dyson Ltd, Malmesbury, UK). These sensors continuously recorded particulate concentrations and geolocation with a 1-min temporal resolution.

Participants wore the monitors over a 5-day period per season, with continuous data logging. Compliance was supported through structured spot-check audits, including unannounced field visits to participant homes or places of work. Fieldworkers conducted regular device checks, data offloading, and battery status assessments to ensure protocol adherence and data completeness.

### Ambient monitoring and meteorological context

Ambient PM_2.5_ concentrations were measured at health facilities in each study country using solar powered Clarity Node-S monitors (Clarity Movement Co, Berkeley, USA). Due to prolonged customs delays and low capture rates, ambient data from Mozambique were excluded from ambient–personal analyses. These monitors were selected for their proven durability in field deployments across low-resource settings. Ambient measurements provided background pollution context and supported analyses of personal-ambient correlations.

We retrieved hourly meteorology from NASA's POWER service (derived from MERRA-2) at the native 0.5° × 0.625° grid, extracting the nearest grid cell to each site; data were converted to local time before merging with exposure records. These parameters enabled stratification of exposure metrics by environmental conditions and supported investigation of pollutant transport dynamics.

### Data quality assurance and processing

Prior to field deployment, all personal and ambient monitoring devices underwent co-location calibration against reference-grade particulate monitoring instruments under controlled environmental conditions. Calibration procedures followed the protocol described by Lim et al. (15), which has previously been applied in multi-country African deployments of the same monitoring platform. Co-location exercises were conducted across a range of particulate concentrations to evaluate inter-device consistency and agreement with reference measurements. Calibration coefficients derived from these exercises were applied during data processing. During field deployment, periodic co-location assessments were repeated to identify sensor drift and verify instrument stability. Devices demonstrating persistent performance anomalies, substantial data loss, or evidence of calibration instability were excluded from analysis. Previous evaluation of the deployed monitoring platform demonstrated strong agreement with reference instruments for PM_2.5_ measurement (15), supporting its suitability for personal exposure assessment in low-resource field settings. Monitors showing low data quality or exceptional performance drift were removed from the deployment cycle. PM_2.5_ concentrations were Winsorised at the 1st and 99th percentiles to limit the influence of extreme outliers without discarding plausible high-exposure events. Missing or dropped geolocation points were interpolated using spatiotemporal smoothing algorithms. Data synchronisation was verified using device-internal timestamps. A minimum data completeness threshold of 75% per deployment cycle was enforced; participants with insufficient data were excluded from the analysis.

Geolocation data were used to classify exposure periods into binary microenvironments: “home” and “away from home”. A geofencing algorithm (radius 50 m) delineated residential locations, and activity segments were categorised using spatial clustering and speed thresholds. The 50 m residential geofence was selected to accommodate expected GPS positional uncertainty, household compound size, and short-distance movement around residential structures in the study settings.

Microenvironment labels were validated through fieldworker-confirmed household coordinates. The away-from-home category should be interpreted as a composite exposure context rather than a specific microenvironment. It may include transport, market attendance, occupational activity, social visits, and other non-residential settings. The available GPS and questionnaire data did not permit robust sub-classification into transport, work, market, or other activity spaces; therefore, source attribution within away-from-home periods is interpreted cautiously. Diurnal activity phases—morning, midday, and evening—were used to map exposure trends to culturally structured routines such as cooking, market attendance, and work-related mobility.

### Statistical analyses

Personal PM_2.5_ exposures were summarised as daily median and upper-quartile “peak” (mean of the top 25% minute-level values). Temporal patterns were described using stratified medians and smoothed visualisations (locally weighted curves), with exposure variability disaggregated by country/site, season (dry/wet), and microenvironment (home vs. away). For each person-day we reported WHO 2021 24-h exceedance (>15 µg/m^3^) with 95% Wilson CIs.

Associations between exposure and structural determinants were examined via stratified statistical assessments. To aid interpretation without over-modelling, we complemented these summaries with non-parametric contrasts (Wilcoxon for binary splits; Kruskal–Wallis for multi-category factors with Dunn–Holm *post-hoc* where relevant) and distributional equity metrics: Gini coefficients (within-country dispersion) and the Concentration Index (direction and magnitude of pro-poor vs. pro-rich concentration). Wealth gradients were further described using Poverty Probability Index quintiles (Q1–Q5) and Spearman's *ρ*. We explored non-linear wealth–exposure patterning using a semiparametric smooth of Poverty Probability Index in a log-scale model of daily exposure, with fixed effects for country/season and a participant-level random intercept.

We assessed ambient–personal correlations by pairing concurrent hourly medians of personal and ambient PM_2.5_. We fit ordinary least squares models on log_10_–log_10_ scales and reported the slope and adjusted *R*^2^. Building on observed heterogeneity in ambient–personal PM_2.5_ relationships, we examined the role of wind speed and direction using pollution roses.

For all analyses, daily exposure is defined as the median of 1-min PM_2.5_ concentrations over a participant-day; “peak” exposure is defined as the mean of the upper-quartile (≥75th percentile) minute-level concentrations within that day (Qmean), selected to characterise sustained high-intensity episodes while reducing sensitivity to single-minute spikes. The episode-intensity index was calculated as peak-Qmean divided by the daily median PM_2.5_ concentration. Values above 1 indicate the extent to which sustained upper-quartile concentrations exceeded the daily central tendency. All analyses were performed in R 4.4.0. Results were validated through reproducible workflows archived in shared repositories.

These analytical components were designed to operationalise an integrated exposure assessment framework, capturing interactions between climatic variability, mobility patterns, and structural determinants, and enabling both descriptive and distributional characterisation of exposure relevant to maternal health contexts. All analyses were pre-specified based on study objectives to minimise data-driven inference.

## Results

### Cohort profile

We enrolled 343 women across The Gambia (*n* = 160), Kenya (*n* = 105), and Mozambique (*n* = 78) (Table 1). Monitoring spanned 328 calendar days and generated 3,190 person-days of observation. Across the cohort, the median age was 26.3 years (IQR: 22.1–31.4), with household reliance on polluting cooking fuels (biomass or charcoal) reported in 87% of households. Most participants (63%) lived in homes classified as having minimal ventilation potential, exceeding 70% in rural areas.

**Table 1:** PRECISE air quality sub-study population demographics.

Characteristic	Overall	The Gambia	Kenya	Mozambique
	*N* = 343	*N* = 160	*N* = 105	*N* = 78
Monitoring Period Start	9-Mar-2022	31-Mar-2022	9-Mar-2022	4-May-2022
Monitoring Period End	31-Jan-2023	31-Jan-2023	13-Dec-2022	11-Oct-2022
Age	26.3 (5.8)	26.5 (5.6)	28.7 (5.2)	22.6 (5.2)
Wealth Index	46 (20)	36 (21)	58 (13)	49 (12)
Extreme Poverty Probability	14 (11)	21 (8)	5 (7)	10 (9)
Parity				
0	81 (24%)	22 (14%)	21 (20%)	38 (52%)
1	75 (22%)	30 (19%)	30 (29%)	15 (21%)
2	60 (18%)	22 (14%)	25 (24%)	13 (18%)
3	46 (14%)	29 (18%)	14 (13%)	3 (4.1%)
4+	76 (22%)	57 (36%)	15 (14%)	4 (5.5%)
Occupation				
Business	13 (3.8%)	0 (0%)	13 (12%)	0 (0%)
Factory	2 (0.6%)	0 (0%)	2 (1.9%)	0 (0%)
Housewife	248 (72%)	141 (88%)	51 (49%)	56 (72%)
Informal—Employment	14 (4.1%)	0 (0%)	14 (13%)	0 (0%)
Market trader	20 (5.8%)	6 (3.8%)	14 (13%)	0 (0%)
Other (specify)	10 (2.9%)	8 (5.0%)	2 (1.9%)	0 (0%)
Professional	11 (3.2%)	3 (1.9%)	8 (7.6%)	0 (0%)
Student	20 (5.8%)	2 (1.3%)	1 (1.0%)	17 (22%)
Missing	5 (1.5%)	0 (0%)	0 (0%)	5 (6.4%)
Education				
Higher	27 (7.9%)	12 (7.5%)	15 (14%)	0 (0%)
None	101 (29%)	93 (58%)	6 (5.7%)	2 (2.6%)
Primary	101 (29%)	19 (12%)	59 (56%)	23 (29%)
Secondary	109 (32%)	36 (23%)	25 (24%)	48 (62%)
Missing	5 (1.5%)	0 (0%)	0 (0%)	5 (6.4%)
Household ventilation				
High air exchange	123 (36%)	82 (51%)	31 (30%)	10 (13%)
Minimal air exchange	216 (63%)	75 (47%)	74 (70%)	67 (86%)
Moderate air exchange	2 (0.6%)	2 (1.3%)	0 (0%)	0 (0%)
Missing	2 (0.6%)	1 (0.6%)	0 (0%)	1 (1.3%)
Cooking fuel				
Battery	3 (0.9%)	0 (0%)	0 (0%)	3 (3.8%)
Biomass	167 (49%)	119 (74%)	26 (25%)	22 (28%)
Coal	131 (38%)	39 (24%)	49 (47%)	43 (55%)
Electricity	8 (2.3%)	0 (0%)	0 (0%)	8 (10%)
Gas	27 (7.9%)	0 (0%)	27 (26%)	0 (0%)
Kerosene	3 (0.9%)	0 (0%)	3 (2.9%)	0 (0%)
Don't know	2 (0.6%)	1 (0.6%)	0 (0%)	1 (1.3%)
None	2 (0.6%)	1 (0.6%)	0 (0%)	1 (1.3%)
Residence				
Rural	141 (41%)	106 (66%)	10 (9.5%)	25 (32%)
Urban	190 (55%)	50 (31%)	92 (88%)	48 (62%)
Missing	12 (3.5%)	4 (2.5%)	3 (2.9%)	5 (6.4%)
Environmental				
Personal PM_2.5_ mean (µg/m^3^)	31 (21)	32 (21)	32 (22)	25 (16)
Personal PM_2.5_ median (µg/m^3^)	12.1 (8.7)	14.4 (11.3)	8.7 (2.7)	12.0 (6.0)
Personal PM_2.5_ peak (µg/m^3^)	91 (71)	93 (64)	105 (84)	67 (60)
Temperature mean (°C)	25.8 (3.1)	27.7 (1.8)	26.1 (1.3)	21.3 (2.6)
Temperature median (°C)	24.89 (2.90)	26.78 (1.30)	25.11 (1.15)	20.70 (2.60)
Temperature peak (°C)	31.5 (4.4)	34.3 (3.3)	31.0 (1.9)	26.3 (3.6)
Humidity mean (%)	71 (11)	68 (13)	75 (5)	72 (10)
Humidity median (%)	75 (12)	72 (15)	81 (5)	74 (11)
Precipitation mean (mm)	0.17 (0.20)	0.26 (0.23)	0.12 (0.13)	0.04 (0.09)
Wind speed mean (m/s)	4.07 (0.89)	3.55 (0.51)	4.79 (0.76)	4.15 (1.00)
Ambient PM_2.5_ mean (µg/m^3^)	6.3	5.4 (1.5)	7.7 (1.8)	NA
Ambient PM_2.5_ median (µg/m^3^)	5.5	4.4 (1.4–7.8)	6.2 (4.2–4.4)	NA
Ambient PM_2.5_. Peak (µg/m^3^)	11.5	10.6 (3.8)	12.7 (2.5)	NA

### Cross-country and seasonal variability in personal PM_2.5_ exposure

Participant-level exposure summaries are shown in Table 2. Daily median PM_2.5_ differed significantly by country (*p* < 0.001), highest in Mozambique (10.9 µg/m^3^), followed by The Gambia (10.4 µg/m^3^) and Kenya (8.1 µg/m^3^). Mean and peak-Qmean exposures demonstrated similar cross-site heterogeneity.

**Table 2:** Participant-level daily personal PM_2.5_ by country.

	Median (IQR)					
Exposure metric	*N*	Overall	The Gambia	Kenya	Mozambique	*p*-value^2^
		*N* = 343^1^	*N* = 160^1^	*N* = 105^1^	*N* = 78^1^	
Median PM_2.5_	343	9.8 (7.4, 12.6)	10.4 (7.6, 14.7)	8.1 (6.8, 9.8)	10.9 (9.3, 13.5)	*p* < 0.001
Mean PM_2.5_	343	23.9 (17.3, 37.7)	27.2 (18.4, 39.7)	25.0 (18.9, 38.3)	19.3 (14.8, 26.0)	*p* = 0.001
Peak PM_2.5_ Qmean	343	65.5 (39.9, 104.1)	69.3 (42.7, 110.9)	76.5 (49.7, 127.2)	42.9 (30.3, 71.3)	*p* < 0.001

1 Median (Q1, Q3).

2 Kruskal–Wallis rank sum test.

Marked right-skew was evident in all settings: daily means substantially exceeded medians—27.2 vs. 10.4 µg/m^3^ in The Gambia, 25.0 vs. 8.1 µg/m^3^ in Kenya, and 19.3 vs. 10.9 µg/m^3^ in Mozambique (Table 2)—indicating that short-duration high concentrations exerted disproportionate influence on overall exposure burden, supporting the use of median concentrations in subsequent analyses.

**Figure 1:**
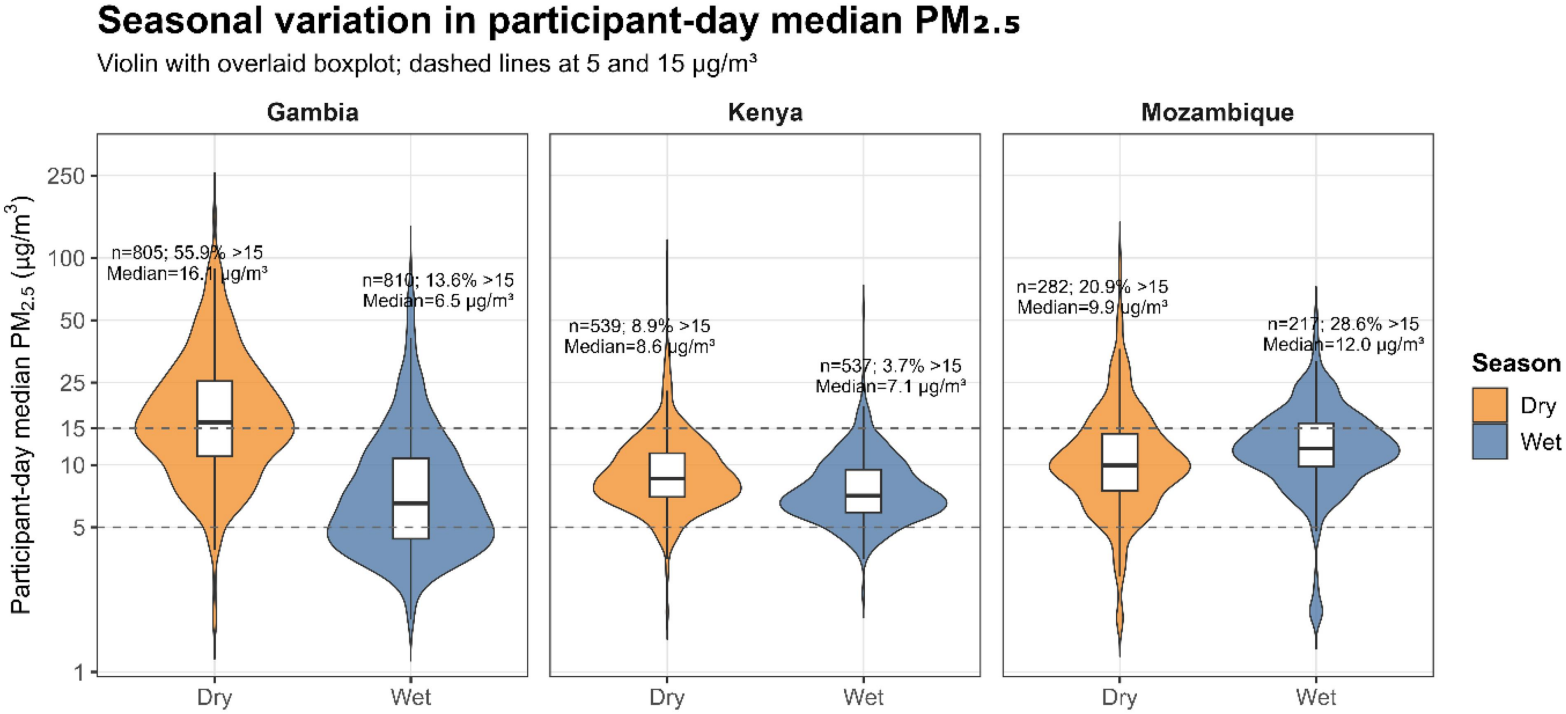
Seasonal patterning of personal PM_2.5_ exposure across three sub-Saharan African settings. Seasonal variation in participant-day median PM_2.5_ concentrations across study settings. Violin plots with embedded boxplots show participant-day median PM_2.5_ concentrations during locally defined dry and wet seasons. In The Gambia, the dry season broadly corresponds to the Harmattan period (November–May), while the wet season corresponds to the rainy season (June–October). In Kenya and Mozambique, dry and wet seasons were defined according to local rainfall and meteorological patterns. Dashed horizontal lines indicate the WHO annual (5 µg/m^3^) and 24-h (15 µg/m^3^) PM_2.5_ guideline values. Annotations indicate the number of participant-days, percentage of participant-days exceeding the WHO 24-h guideline, and the country-season median concentration.

Seasonal contrasts were pronounced (Table 3; Figure 1A), illustrating the upward shift in exposure distributions during dry-season periods across all sites. Across countries combined, dry-season medians (11.5 µg/m^3^) exceeded wet-season medians (7.3 µg/m^3^; *p* < 0.001).

**Table 3:** Participant–season personal PM_2.5_ (dry vs. wet).

	Median (IQR)				
Exposure metric	*N*	Overall	Dry	Wet	*p*-value^2^
		*N* = 555^1^	*N* = 288^1^	*N* = 267^1^	
median_PM25	555	9.7 (6.8, 14.2)	11.5 (8.6, 17.5)	7.3 (5.5, 10.4)	*p* < 0.001
mean_PM25	555	23.6 (15.8, 38.3)	27.9 (18.5, 41.1)	20.0 (13.9, 31.7)	*p* < 0.001
peak_PM25_Qmean	555	61.1 (36.2, 108.8)	71.1 (42.4, 120.6)	55.2 (32.7, 96.6)	*p* = 0.001

1 Median (Q1, Q3).

2 Wilcoxon rank sum test.

**Figure 2:**
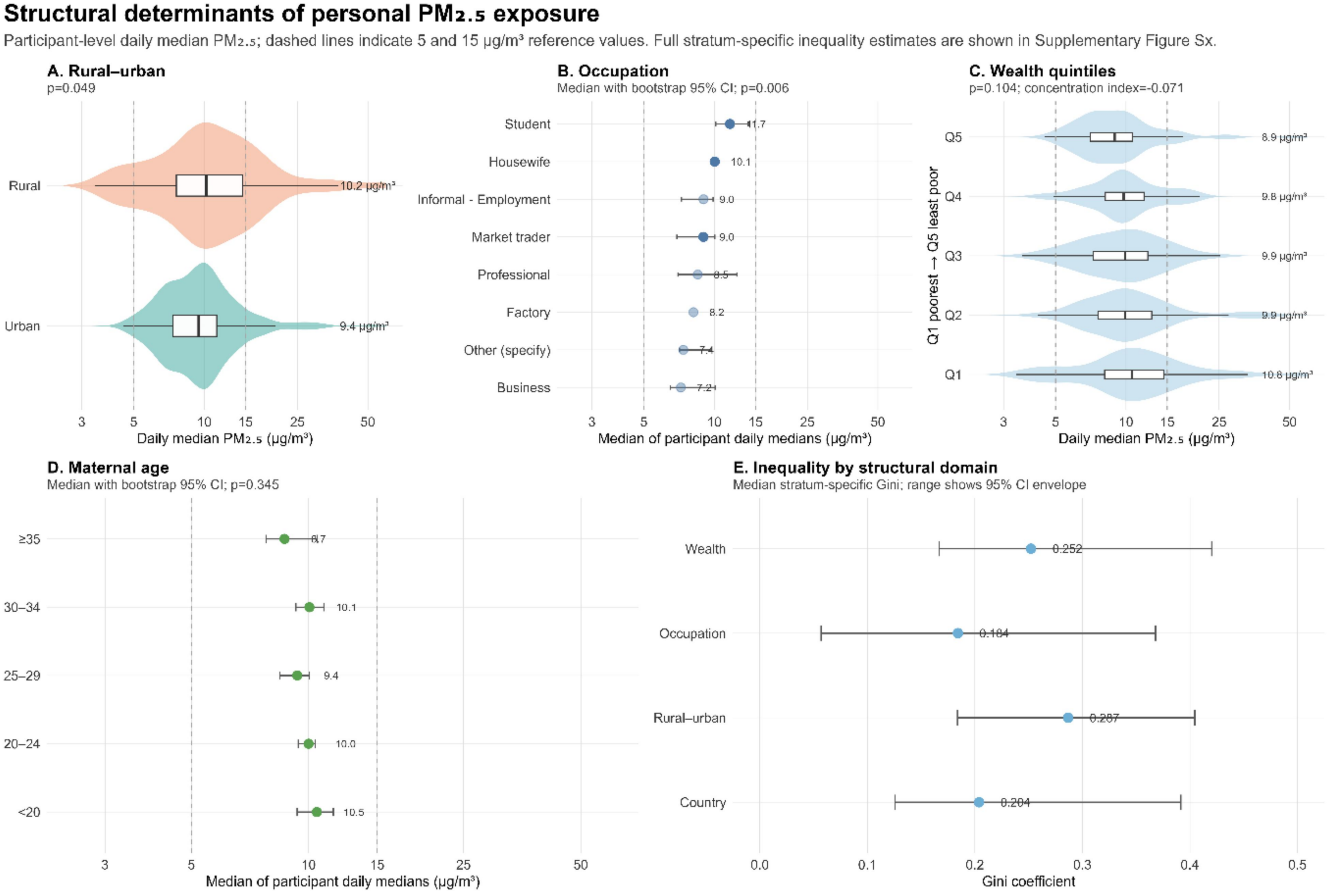
Seasonal distribution of WHO PM_2.5_ guideline exceedances. Percentage of person-days exceeding the WHO 2021 24-h PM_2.5_ guideline (>15 µg/m^3^), stratified by country and season. Bars represent proportions with 95% Wilson confidence intervals. These findings illustrate strong seasonal and geographic heterogeneity in exposure exceedance patterns.

WHO 2021 24-h guideline exceedances (>15 µg/m^3^) were strongly seasonal and site-specific (Table 4; Figure 2; Supplementary Figure S1). The Gambia exhibited the largest dry–wet contrast (55.9% vs. 13.6%), consistent with seasonal dust loading during Harmattan months. Kenya showed comparatively low exceedance proportions (8.9% dry; 3.7% wet). In contrast, Mozambique demonstrated elevated exceedances in both seasons, with a higher proportion during the wet season (28.6% vs. 20.9%), warranting further examination of local emission sources, meteorological conditions, and activity patterns. Monthly patterns further demonstrated climate sensitivity.

**Table 4:** WHO daily health guideline exceedance by site and season (person-days).

Country	Season	Total Person Days	Person Days Exceeding Threshold	Exceedance, % (95% CI)
The Gambia	Dry	805	450	55.9 (52.4–59.4)
The Gambia	Wet	810	110	13.6 (11.3–16.2)
Kenya	Dry	539	48	8.9 (6.7–11.7)
Kenya	Wet	537	20	3.7 (2.4–5.8)
Mozambique	Dry	282	59	20.9 (16.4–26.2)
Mozambique	Wet	217	62	28.6 (22.8–35.2)

In Mozambique, wet-season exceedance was higher than dry-season exceedance despite the expected particle-removal effect of rainfall. This pattern is interpreted cautiously because Mozambique had a smaller number of monitored person-days and because personal exposure may reflect localised wet-season combustion, humidity, and activity-pattern effects not captured by regional seasonal assumptions.

To quantify episodic dominance, we calculated an episode-intensity index (peak-Qmean ÷ median). The cohort-wide median index was 4.31 (IQR: 2.50–9.42), indicating that upper-quartile peaks were typically 331% higher than daily central tendency (Supplementary Table M3), reinforcing the strongly right-tailed exposure distributions.

**Figure 3:**
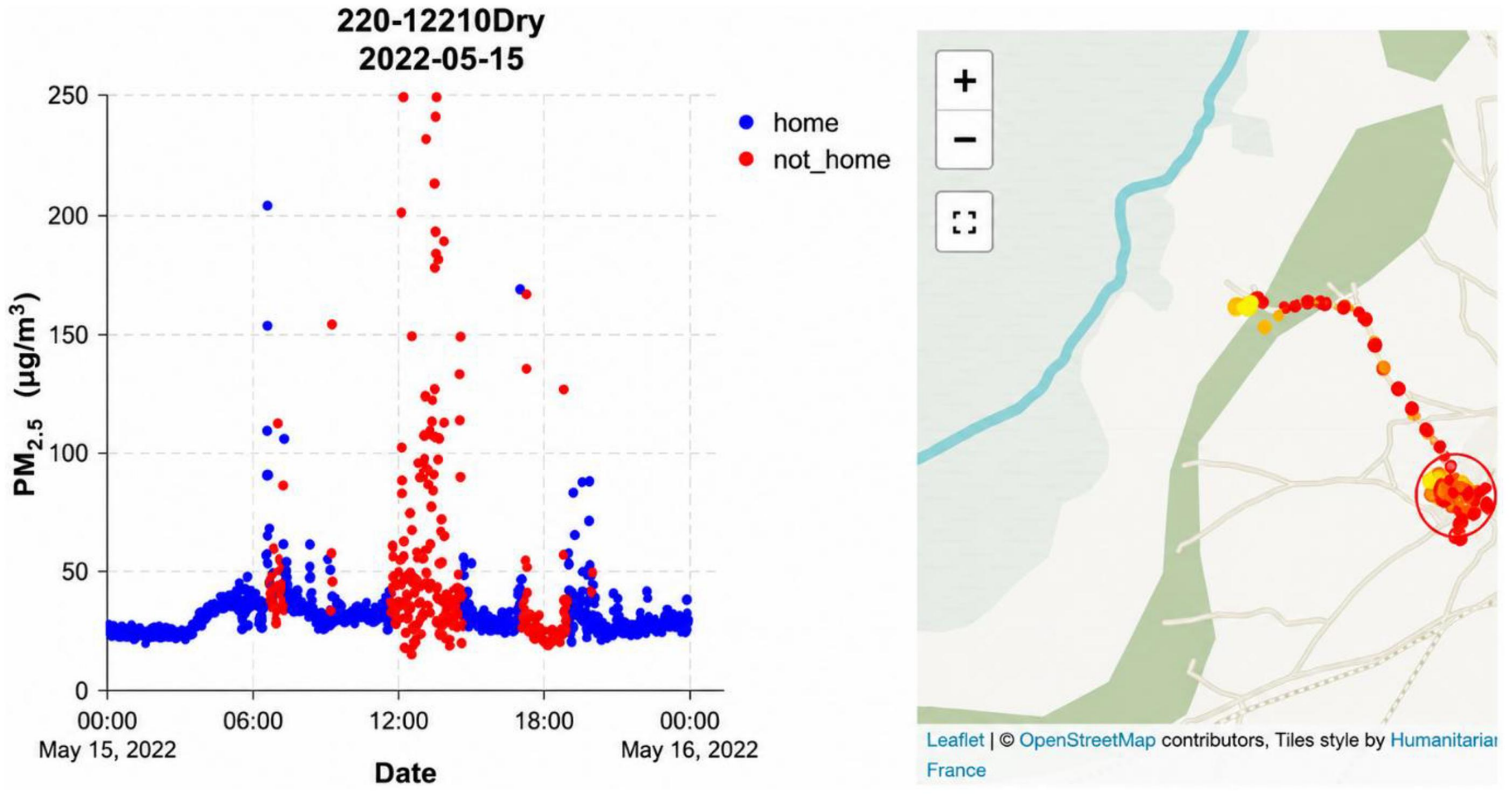
Diurnal variation in personal PM_2.5_ exposure across study settings. Hourly median PM_2.5_ concentrations aggregated across participant-days, stratified by country. Curves represent smoothed median exposure profiles, highlighting consistent site-specific peak exposure windows associated with daily activity patterns.

Diurnal patterns revealed reproducible site-specific peak windows (Figure 3). In The Gambia, peak hour clustered around 12:00–13:00, whereas Kenya and Mozambique showed later peaks (∼16:00). Importantly, peak timing did not differ materially between seasons, suggesting that daily activity patterns rather than purely meteorological forcing structured exposure timing.

### Structural patterning of personal PM_2.5_ exposure

**Figure 4:**
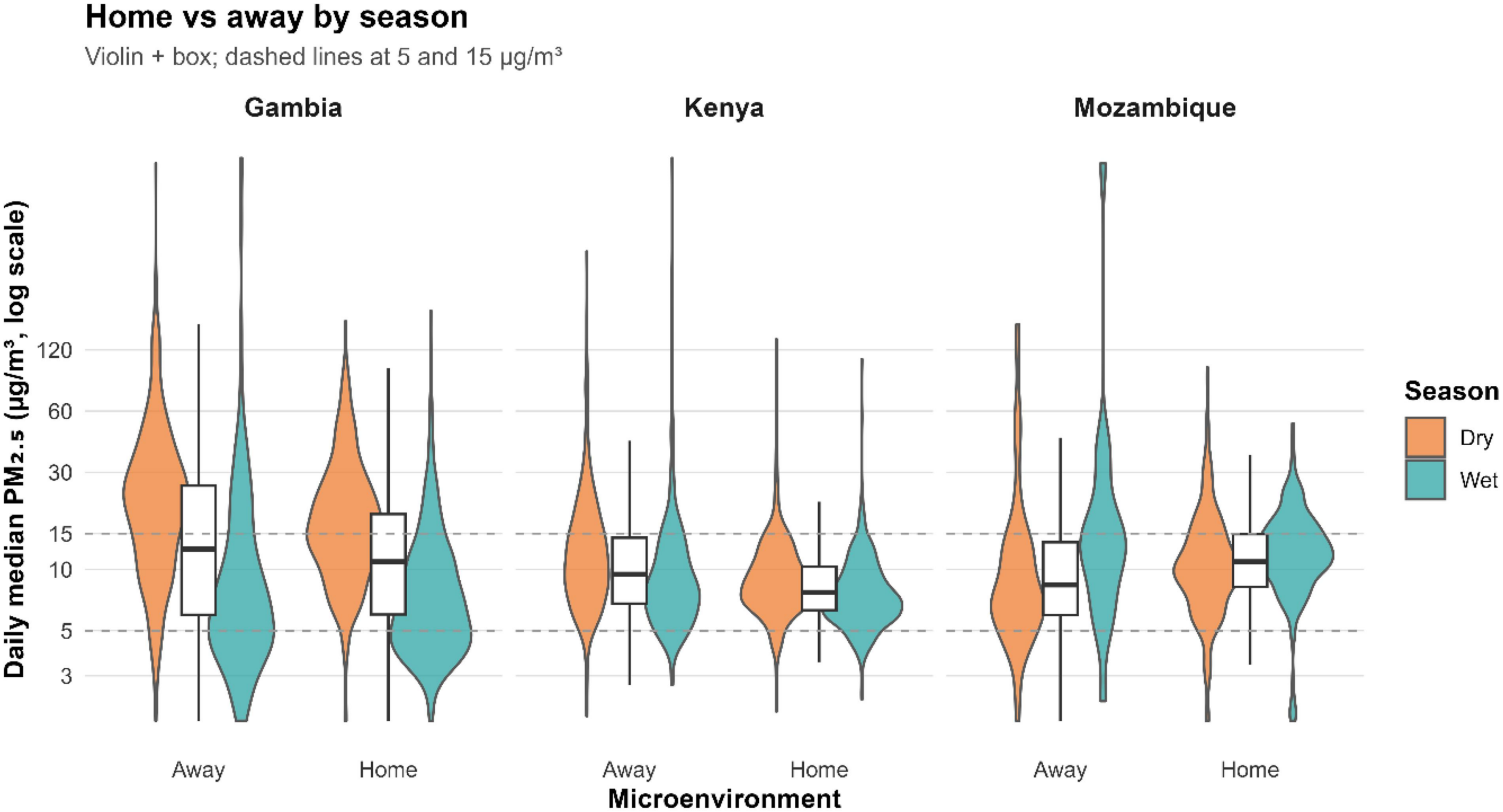
Structural determinants of personal PM_2.5_ exposure. Participant-level daily median PM_2.5_ concentrations are shown across structural and demographic determinants. Panel **A** shows rural–urban differences using violin plots with embedded boxplots; annotated values indicate group medians. Panel **B** shows occupation-specific median exposure estimates with bootstrap 95% confidence intervals. Panel **C** shows exposure distributions across Poverty Probability Index wealth quintiles, from Q1 poorest to Q5 least poor. Panel **D** shows maternal age-band-specific median exposure estimates with bootstrap 95% confidence intervals. Panel E summarises exposure inequality across structural domains using Gini coefficients derived from participant-level daily median PM_2.5_ values; horizontal lines represent the 95% confidence interval envelope across stratum-specific estimates. Dashed vertical reference lines in Panels A–D indicate 5 µg/m^3^ and 15 µg/m^3^. CI, confidence interval; PM_2.5_, fine particulate matter.

Structural gradients are summarised in Figure 4 and Tables 5–9.

**Table 5:** Participant-level PM_2.5_ by rural vs. urban residence.

	Median (IQR)				
Exposure metric	*N*	Overall	Rural	Urban	*p*-value^2^
		*N* = 331^1^	*N* = 141^1^	*N* = 190^1^	
median_PM25	331	9.8 (7.4, 12.7)	10.2 (7.6, 14.7)	9.4 (7.3, 11.3)	*p* = 0.049
mean_PM25	331	23.9 (17.3, 37.7)	24.3 (17.8, 38.4)	23.7 (17.0, 36.9)	*p* = 0.644
peak_PM25_Qmean	331	66.0 (40.1, 104.1)	63.9 (40.4, 104.7)	66.7 (39.5, 104.0)	*p* = 0.735

1 Median (Q1, Q3).

2 Wilcoxon rank sum test.

**Table 6:** Participant-level PM_2.5_ by occupation.

	Median (IQR)									
Exposure metric	*N*	Business	Factory	Housewife	Informal—Employment	Market trader	Other (specify)	Professional	Student	*p*-value^2^
		*N* = 13^1^	*N* = 2^1^	*N* = 248^1^	*N* = 14^1^	*N* = 20^1^	*N* = 10^1^	*N* = 11^1^	*N* = 20^1^	
Median PM_2.5_	338	7.2 (6.5, 10.1)	8.2 (7.0, 9.3)	10.1 (7.8, 13.5)	9.0 (7.0, 9.9)	9.0 (6.7, 10.5)	7.4 (7.1, 9.7)	8.5 (7.0, 12.5)	11.7 (9.9, 14.5)	*p* = 0.006
Mean PM_2.5_	338	21.6 (19.5, 38.2)	24.3 (19.3, 29.2)	25.0 (17.5, 38.4)	28.1 (20.7, 46.0)	21.2 (15.1, 39.7)	19.6 (12.9, 21.9)	22.8 (15.1, 28.1)	25.6 (20.0, 37.1)	*p* = 0.395
Peak PM_2.5_ Qmean	338	68.3 (55.7, 120.3)	70.7 (54.3, 87.1)	66.1 (40.0, 108.5)	87.4 (61.3, 149.3)	61.9 (30.8, 109.1)	48.8 (30.0, 61.3)	45.7 (37.9, 76.5)	71.6 (53.1, 99.3)	*p* = 0.305

1 Median (Q1, Q3).

2 Kruskal–Wallis rank sum test.

**Table 7:** Participant-level PM_2.5_ by cooking fuel type.

	Median (IQR)										
Exposure metric	N	Overall	Battery	Biomass	Coal	Don't know	Electricity	Gas	Kerosene	None	*p*-value^2^
		*N* = 343^1^	*N* = 3^1^	*N* = 167^1^	*N* = 131^1^	*N* = 2^1^	*N* = 8^1^	*N* = 27^1^	*N* = 3^1^	*N* = 2^1^	
median_PM25	343	9.8 (7.4, 12.6)	10.5 (9.3, 11.5)	10.1 (7.7, 13.7)	9.6 (7.3, 12.5)	8.9 (5.2, 12.6)	10.9 (10.0, 12.8)	7.6 (6.7, 10.5)	7.5 (5.8, 9.0)	6.2 (1.8, 10.5)	*p* = 0.050
mean_PM25	343	23.9 (17.3, 37.7)	18.5 (15.5, 18.7)	25.5 (18.0, 42.8)	23.7 (17.2, 31.9)	22.2 (17.6, 26.9)	18.2 (13.8, 51.9)	21.3 (15.5, 28.7)	14.9 (10.9, 28.9)	16.5 (12.9, 20.0)	*p* = 0.121
peak_PM25_Qmean	343	65.5 (39.9, 104.1)	41.8 (36.2, 45.4)	71.0 (42.4, 127.7)	66.2 (39.6, 87.8)	42.8 (35.6, 50.1)	39.9 (28.4, 154.4)	60.3 (43.7, 91.4)	32.7 (26.3, 93.0)	46.9 (41.0, 52.7)	*p* = 0.314

1 Median (Q1, Q3).

2 Kruskal–Wallis rank sum test.

**Table 8:** Participant-level personal PM_2.5_ by wealth (PPI quintiles).

	Median (IQR)							
Exposure metric	N	Overall	Q1	Q2	Q3	Q4	Q5	*p*-value^2^
		*N* = 338^1^	*N* = 68^1^	*N* = 68^1^	*N* = 68^1^	*N* = 67^1^	*N* = 67^1^	
Median PM2.5	338	9.8 (7.4, 12.7)	10.8 (8.1, 14.7)	9.9 (7.4, 13.0)	9.9 (7.1, 12.4)	9.8 (8.0, 12.1)	9.0 (7.0, 10.9)	*p* = 0.104
Mean PM2.5	338	24.3 (17.4, 38.2)	28.3 (19.7, 41.7)	25.5 (17.7, 43.1)	25.7 (17.4, 38.9)	23.1 (15.9, 33.1)	21.4 (15.5, 29.9)	*p* = 0.044
Peak PM2.5 QMean	338	65.9 (40.3, 104.7)	71.1 (44.8, 114.7)	75.7 (42.7, 126.9)	66.7 (41.4, 120.9)	50.0 (37.4, 100.5)	56.8 (36.7, 82.4)	*p* = 0.103

1 Median (Q1, Q3).

2 Kruskal–Wallis rank sum test.

**Table 9:** Participant–season personal PM_2.5_ by wealth (PPI quintiles).

	Median (IQR)							
Exposure metric	*N*	Overall	Q1	Q2	Q3	Q4	Q5	*p*-value^2^
		*N* = 549^1^	*N* = 116^1^	*N* = 110^1^	*N* = 105^1^	*N* = 108^1^	*N* = 110^1^	
Season	549							*p* = 0.989
Dry	NA	285 (52%)	58 (50%)	59 (54%)	55 (52%)	56 (52%)	57 (52%)	
Wet	NA	264 (48%)	58 (50%)	51 (46%)	50 (48%)	52 (48%)	53 (48%)	
Median PM_2.5_	549	9.7 (6.7, 14.2)	10.8 (6.6, 19.3)	9.8 (6.9, 14.8)	9.2 (6.8, 12.5)	9.7 (6.9, 13.4)	8.2 (6.6, 12.6)	*p* = 0.080
Mean PM_2.5_	549	23.7 (15.9, 38.3)	26.5 (16.6, 42.3)	26.1 (17.2, 42.3)	25.3 (17.3, 38.3)	20.9 (15.4, 38.8)	20.3 (15.5, 29.9)	*p* = 0.029
Peak PM_2.5_ QMean	549	61.3 (36.9, 109.3)	70.0 (36.5, 119.5)	71.7 (41.0, 130.5)	72.2 (41.0, 115.1)	49.3 (35.4, 106.1)	56.1 (35.6, 82.3)	*p* = 0.027

1 *n* (%); Median (Q1, Q3).

2 Pearson's Chi-squared test; Kruskal–Wallis rank sum test.

Daily median exposures were modestly higher among rural participants (10.2 µg/m^3^) compared with urban participants (9.4 µg/m^3^; *p* = 0.049; Table 5; Figure 4A). Mean and peak-Qmean differences were not significant, indicating that rural–urban contrasts influenced central tendency rather than episodic intensity.

Median exposures differed significantly by occupation (*p* = 0.006; Table 6; Figure 4B). Students (11.7 µg/m^3^) and housewives (10.1 µg/m^3^) exhibited higher central tendencies, whereas business and “other” categories showed lower medians. Mean and peak-Qmean metrics did not differ significantly across occupational groups, suggesting that occupational gradients reflect sustained baseline exposure rather than disproportionate episodic spikes.

Wealth gradients were modest but directionally consistent. At the participant level, poorer quintiles exhibited higher mean exposures (*p* = 0.044; Table 8), with weaker gradients for medians (*p* = 0.104). When stratified by season (Table 9), dry-season disparities strengthened and peak-Qmean differed significantly across wealth quintiles (*p* = 0.027), indicating seasonal amplification of wealth-related inequality. Rank-based gradients were further summarised using the Concentration Index (Figure 4C).

Exposure dispersion within sites was substantial and heterogeneous. Gini coefficients for participant-level daily medians were highest in The Gambia (0.343; 95% CI: 0.285–0.392), intermediate in Mozambique (0.204; 0.145–0.272), and lowest in Kenya (0.153; 0.124–0.181) (Supplementary Table M4). These estimates confirm that exposure burden is disproportionately concentrated among a minority of individuals within each setting. The extent of within-country exposure inequality is further illustrated in Figure 4E, highlighting substantial dispersion in The Gambia relative to Kenya and Mozambique.

No significant associations were observed between maternal age and exposure (Figure 4D; *p* = 0.345), indicating that structural and environmental conditions, rather than age *per se*, drive observed exposure heterogeneity.

### Microenvironmental and fuel-specific determinants

**Figure 5:**
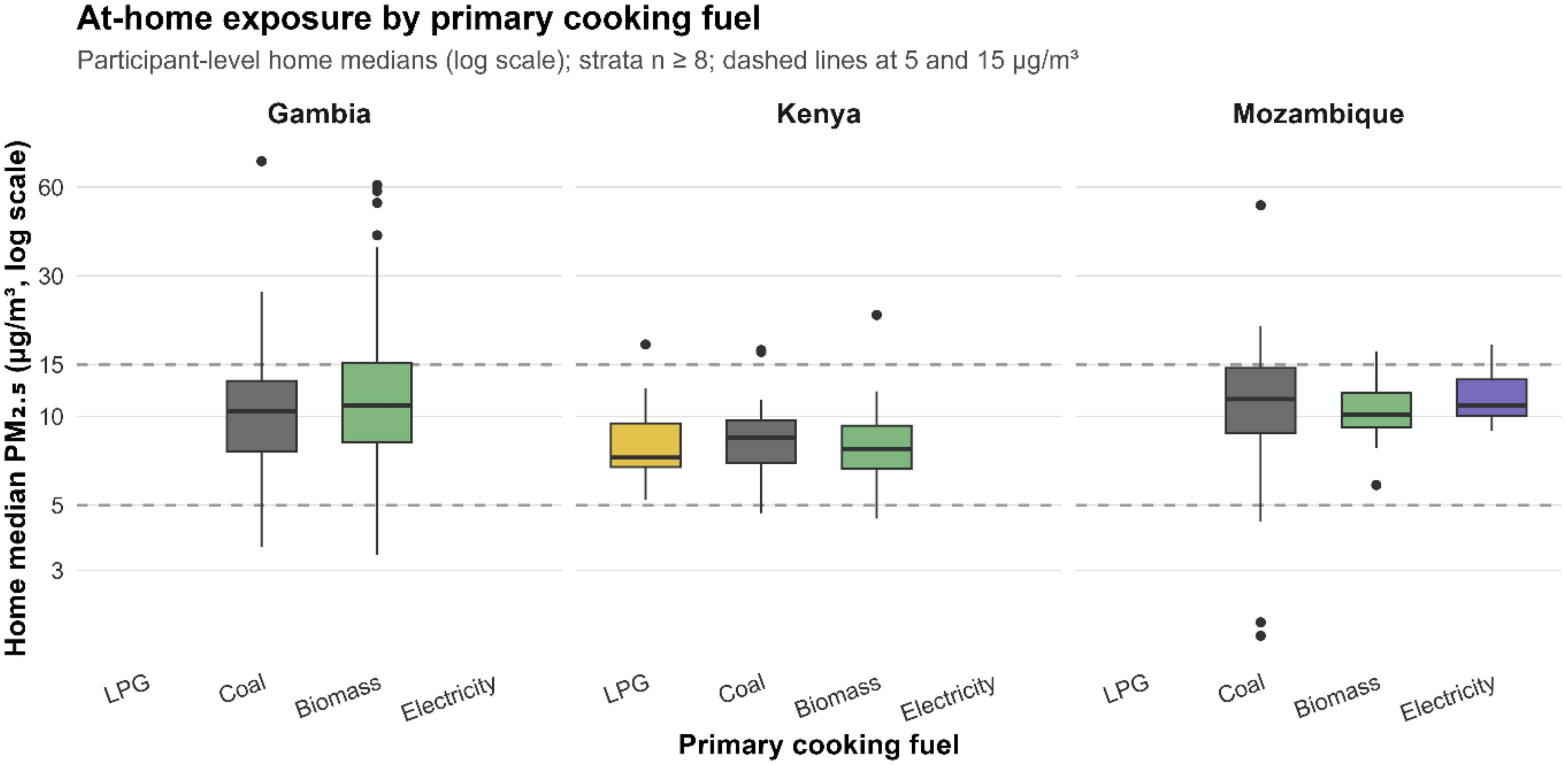
Illustrative personal PM₂․₅ and mobility for one participant in May 2022. Left: minute-level personal PM₂․₅ concentrations coloured by microenvironment classification (blue = at home; red = away from home) for one anonymised participant-day. Right: contemporaneous GPS trace for the same day; red clusters (higher concentrations) align with time spent away from home along travel corridors/market areas, whereas orange points near the residence show lower levels. Map shown for illustration; precise locations anonymised. Figure reproduced from original high-resolution analytical outputs; geographic coordinates have been spatially masked to protect participant confidentiality.

Geolocation-enabled classification allowed separation of exposures into home and away contexts. An illustrative participant-day is shown in Figure 5, demonstrating clear temporal alignment between elevated concentrations and away-from-home mobility segments. This example demonstrates the temporal alignment between elevated concentrations and away-from-home mobility segments.

**Figure 6:**
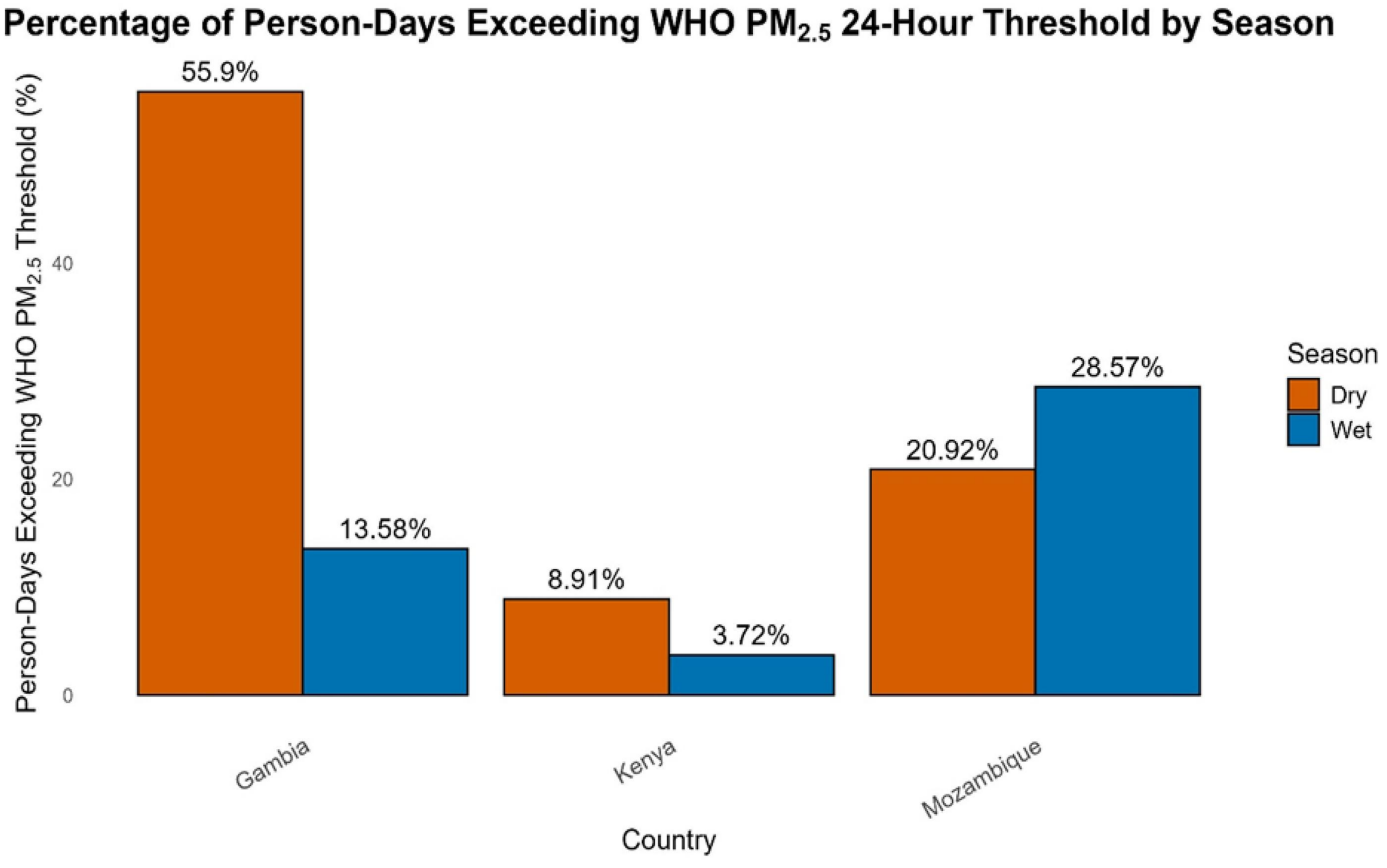
Home vs. away from Home Personal PM_2.5_ Exposure by Season. Daily median PM₂․₅ by microenvironment (Away vs. Home) and season for each country; violins with overlaid boxplots (median line, IQR) on a log scale; dashed lines mark 5 and 15 µg/m^3^ (WHO annual and 24-h guideline, respectively). These findings collectively demonstrate that mobility-linked exposures contribute substantially to daily exposure burden and are not adequately captured by household-based assessments alone.

Across the cohort, exposures were significantly higher away from home. Within-day paired comparisons showed a median difference of +0.8 µg/m^3^ (95% CI: 0.6–1.2; *p* < 0.001) (Figures 6A,B; Figure 8; Supplementary Figure S4). Figure 8 further illustrates the daytime distribution of personal PM₂.₅ exposure by microenvironment, season, and country. Elevations were consistent in Kenya (both seasons) and in The Gambia during the dry season, but not statistically significant in Mozambique, where sample size was smaller and variance greater.

**Figure 7:**
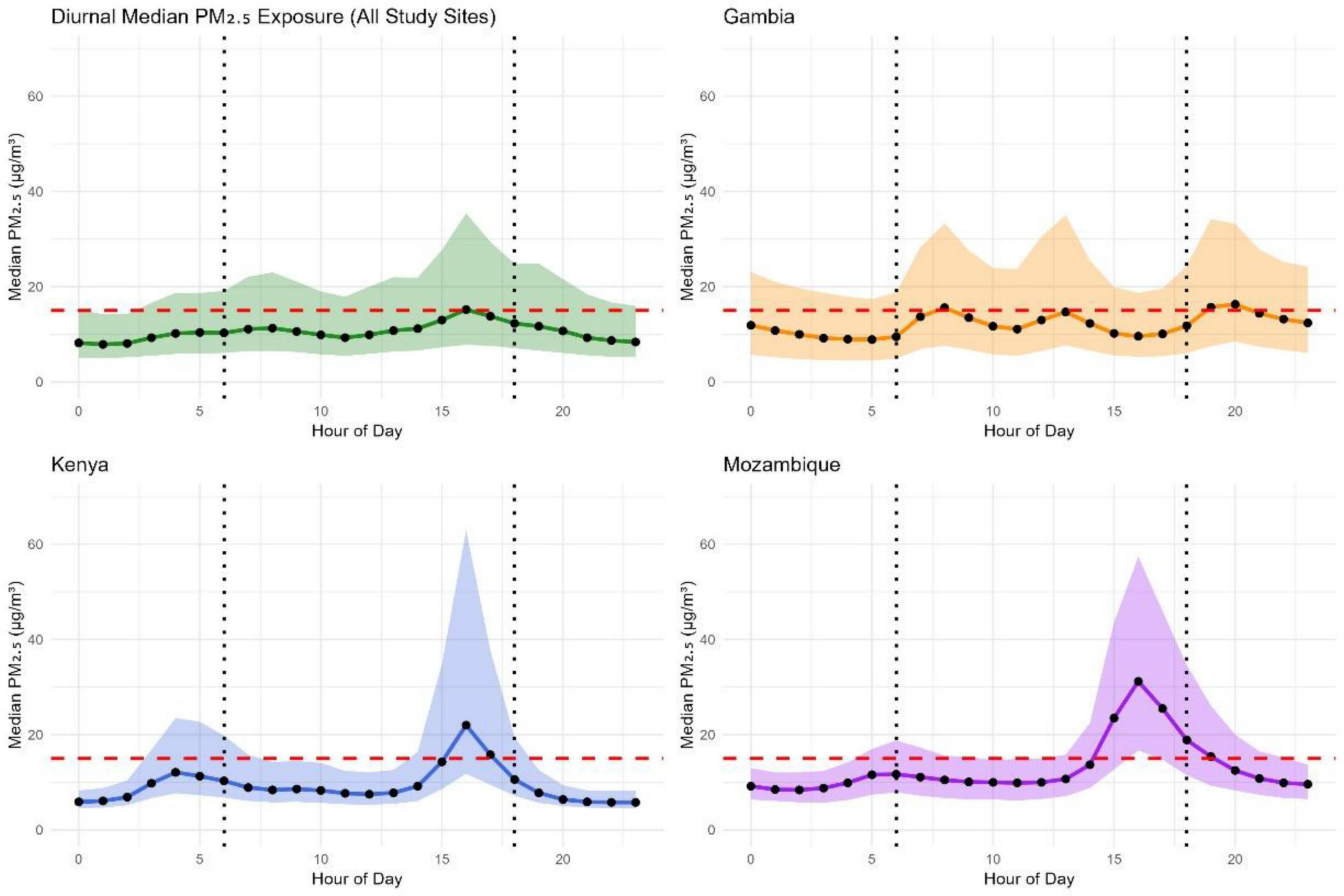
At-home personal PM_2.5_ exposure by primary cooking fuel across study settings. Participant-level daily median PM_2.5_ concentrations measured within the home microenvironment, stratified by primary cooking fuel (LPG, coal, biomass, electricity) and country. Boxplots represent medians and interquartile ranges on a log_10_ scale, with points indicating outliers. Dashed horizontal lines indicate WHO guideline values (5 and 15 µg/m^3^).These distributions show higher at-home median PM_2.5_ concentrations among biomass and coal users than among LPG and electricity users. However, substantial overlap across fuel categories and the presence of elevated values among cleaner-fuel users suggest that household fuel type alone does not fully explain personal exposure, with non-household and episodic sources also likely contributing to exposure variability.

At-home fuel type influenced median exposures (*p* = 0.050; Table 7; Figure 7). Biomass and coal users exhibited higher at-home median PM_2.5_ concentrations than LPG and electricity users. However, substantial overlap across fuel categories and the presence of elevated values among cleaner-fuel users suggest that household fuel type alone does not fully explain personal exposure variability. This pattern reinforces that while household fuel use contributes to baseline exposure, it does not fully capture the distribution of personal exposure, particularly in contexts where mobility and peri-domestic sources are significant.

**Figure 8:**
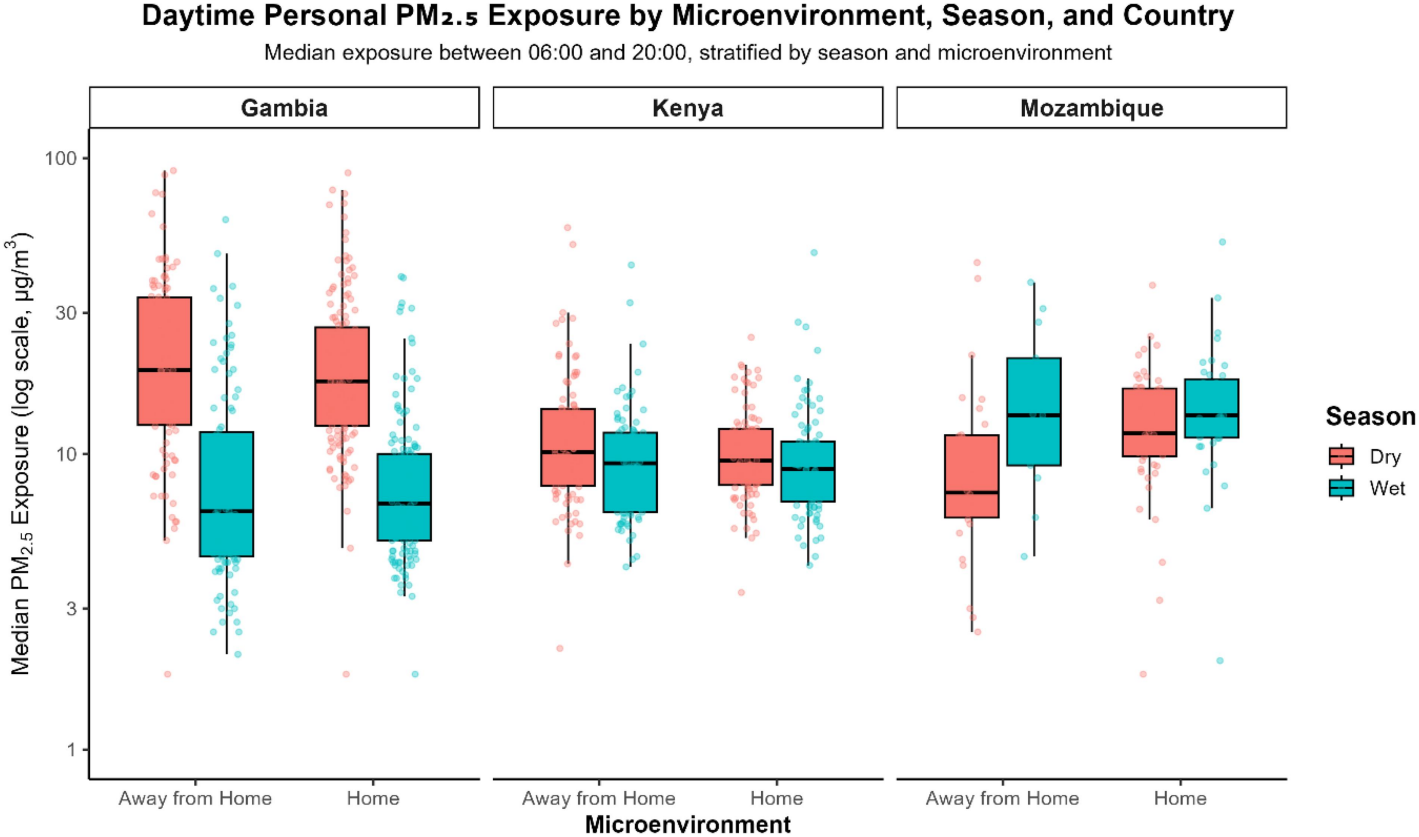
Daytime personal PM_2.5_ exposure by microenvironment, season, and country. Distribution of daytime (06:00–18:00) personal PM_2.5_ concentrations stratified by microenvironment (home vs. away), season (dry vs. wet), and country. Violin plots display distributional spread with embedded boxplots (median and IQR). These results demonstrate consistent elevation of exposure during away-from-home periods across multiple contexts.

In contrast, mean and peak-Qmean exposures did not differ significantly across fuel types (Kruskal–Wallis *p* = 0.121 and *p* = 0.314, respectively), indicating that short-duration high-intensity exposure events occurred even among households using cleaner fuels. This comparison could not be meaningfully evaluated within The Gambia because of the near-universal reliance on biomass fuels. Away-from-home peaks clustered between 12:00 and 16:00 (Figure 5 & Supplementary Figure S4), aligning with travel and market activity windows. Consistent peak timing across seasons further supports behavioural structuring of exposure rather than exclusively meteorological timing.

### Ambient–personal concordance and meteorological context

Ambient–personal relationships were evaluated for The Gambia and Kenya; ambient data capture rates were insufficient to allow robust analysis in Mozambique.

Hourly ambient PM_2.5_ explained a modest proportion of personal exposure variance (adjusted *R*^2^ range: 0.01–0.56). Supplementary Figure S2 illustrates this heterogeneity across sites and seasons, with regression slopes consistently below unity. Concordance was strongest in Kenyan wet-season sites and weakest in The Gambia, consistent with greater contributions from indoor and peri-domestic sources.

Log–log regression slopes were generally <1, indicating systematic underestimation of personal exposure by ambient proxies.

Wind-rose diagnostics (Supplementary Figure S3) showed predominantly southerly flow in Kenya and south-westerly flow in The Gambia. Wind-direction analyses were used only as descriptive diagnostics. Limited directional variability, sparse fixed-site monitoring, and the absence of source-specific chemical composition data prevented formal source attribution. Observed exposure patterns are therefore interpreted in relation to plausible regional and local sources, including Harmattan dust in The Gambia, biomass and charcoal combustion, traffic-related emissions, roadside dust, and peri-domestic or market-based combustion. These analyses are therefore interpreted descriptively rather than causally.

## Discussion

These findings provide direct empirical support for hypothesised maternal–newborn health pathways. Episodic PM_2.5_ peaks and sustained short-term personal exposure profiles measured during pregnancy may be relevant to hypothesised maternal cardiovascular and placental stress pathways, although this study did not estimate cumulative exposure across gestation. The observed dominance of short-duration high-intensity exposures and mobility-linked exposure environments suggests that critical windows of vulnerability may not be adequately captured by ambient or household-based exposure models. These exposure characterisations provide a critical empirical foundation for subsequent analyses examining associations between PM_2.5_ exposure and maternal and perinatal outcomes within the PRECISE cohort.

This multi-country, time-resolved characterisation of personal PM_2.5_ exposure in The Gambia, Kenya, and Mozambique demonstrates that exposure in Sub-Saharan Africa is not only spatially and seasonally variable but also structurally patterned by poverty, livelihoods, household energy, and place. Typical daily medians often sit near the WHO 24-h guideline, yet short, high-intensity spikes—captured through the episode-intensity index—elevate daily means and account for a substantial share of exceedance days, particularly during dry seasons.

Four interlinked insights emerge:

### Climate amplification and distributional shape

Seasonal contrasts were pronounced but not uniform across settings. Dry-season amplification in The Gambia aligns with regional dust transport during Harmattan months, when reduced precipitation and altered atmospheric mixing enhance particulate persistence. However, the elevated wet-season exceedance observed in Mozambique underscores that seasonal exposure trajectories are also context-specific.

The higher wet-season exceedance proportion in Mozambique requires direct interpretation because it contrasts with the expected pattern of lower particulate concentrations during rainy periods. Rather than indicating that rainfall generally increases PM_2.5_, this finding suggests that rainfall effects on personal exposure are context dependent. Wet deposition may reduce suspended dust and background particles during or shortly after sustained rainfall; however, intermittent rainfall, high humidity, and poor dispersion can also promote particle accumulation in enclosed or semi-enclosed microenvironments. Hygroscopic growth under humid conditions may increase measured particle mass, while damp biomass or charcoal may burn less efficiently and produce more visible smoke during household or peri-domestic combustion. In the Mozambique study setting, the wet-season exceedance pattern may therefore reflect the interaction of rainfall, humidity, fuel moisture, and behavioural adaptation to rain.

This interpretation is consistent with the personal-exposure nature of the data. Unlike fixed-site ambient monitoring, wearable monitors capture exposure during cooking, sheltering indoors, movement through markets, and other daily activities. Rainy conditions may shift pregnant women's time-activity patterns towards covered or indoor environments where combustion emissions accumulate, while also modifying travel routes and the duration of stays in semi-enclosed communal spaces. The smaller Mozambique sample and shorter monitoring period further caution against interpreting this pattern as a general reversal of rainfall scavenging. Instead, the finding highlights that wet-season exposure in this setting may be driven by localised personal and peri-domestic emissions that are not fully offset by precipitation-related particle removal. We therefore interpret the Mozambique wet-season anomaly as evidence of local exposure complexity rather than as contradiction of the broader role of rainfall in reducing regional particulate loading.

Beyond seasonal magnitude, the divergence between daily medians and means reveals the structural importance of episodic spikes. In all sites, means substantially exceeded medians, indicating right-skewed distributions dominated by short-duration high-concentration events. This distinction is not merely statistical: median-based summaries capture central tendency, but mean and upper-quartile metrics reflect the physiological reality of repeated inflammatory insults that may be relevant for maternal cardiovascular and placental stress pathways.

Diurnal peak timing was stable across seasons, suggesting that behavioural routines—travel, market activity, informal work—structure exposure timing more consistently than meteorology structures exposure occurrence. In this sense, climate amplifies exposure burden, but behaviour determines when and how that burden is experienced.

The observed differences in peak exposure timing between countries are also consistent with locally structured patterns of daily activity and mobility. In The Gambia, the pronounced midday peak between approximately 12:00 and 13:00 coincides with a period of intense daytime activity, including market attendance, social interaction, food preparation, and travel during the hottest part of the day. In contrast, the later afternoon peaks observed in Kenya and Mozambique (approximately 16:00) correspond more closely to the end of standard working periods, school collection, return journeys, roadside commerce, and preparation of the evening meal (8, 16). These activities increase movement through multiple microenvironments and may elevate exposure through interactions with transport emissions, commercial cooking activities, roadside dust, and other community-level sources.

Importantly, these temporal patterns are difficult to explain solely by meteorological forcing. If atmospheric dispersion processes were the dominant determinant, broadly similar diurnal profiles would be expected across the three study settings. Instead, the country-specific timing of exposure maxima suggests that exposure is shaped by the interaction between local emission environments and socially patterned daily behaviours. This interpretation is further supported by our finding that participants experienced higher PM_2.5_ concentrations while away from home than while at home, indicating that routine mobility and time-activity patterns play an important role in determining personal exposure.

Although detailed activity diaries were not collected, GPS-derived mobility data demonstrated substantial daily movement beyond the household environment, supporting the interpretation that personal exposure profiles reflect repeated transitions between microenvironments rather than static residential exposure alone.

### Structural patterning and within-place inequality

Participant daily median exposures were modestly higher in rural than urban settlements, yet mean and peak-Qmean metrics were similar, indicating that rural–urban contrasts primarily affected baseline exposure rather than episodic intensity. This pattern likely reflects differences in household fuel reliance and ventilation alongside varying density of traffic and market emissions in more urbanised contexts.

Structural gradients were evident across occupation and wealth strata. Poorer households and certain livelihood categories experienced higher exposures, and these disparities intensified under seasonal stratification. Within-place dispersion, quantified using Gini coefficients, demonstrated that a minority of individuals shoulder a disproportionate burden of exposure even within the same settlement.

These findings extend prior literature linking poverty and household energy use to pollution exposure by demonstrating that inequality operates not only between rural and urban contexts but within communities. Exposure inequity is therefore not solely a function of geography, but of structural positioning within place.

Maternal age was not independently associated with exposure, reinforcing that structural and environmental determinants—rather than demographic age *per se*—drive observed heterogeneity.

### Beyond the household lens: mobility and peri-domestic complexity

The paired home–away contrasts represent one of the most policy-relevant findings of this work. The median difference of +0.8 µg/m^3^ away from home indicates that mobility-linked environments materially contribute to total daily exposure. Consistent elevations in Kenya (both seasons) and in The Gambia during the dry season demonstrate that household energy choices influence central tendency at home, but mobility-linked, out-of-home microenvironments are major contributors to health-relevant daily exposure in several settings.

Biomass use at home likely contributes not only to indoor concentrations but also to surrounding compounds and shared community spaces, including market cooking stalls and informal trading areas. In The Gambia, background peak alignment with travel corridors suggests that peri-domestic and transport-related emissions may compound household sources.

Clean-fuel use lowers at-home medians but does not eliminate short high-intensity peaks. This finding complicates a purely household-centric mitigation strategy. Interventions confined to domestic fuel transitions will reduce baseline exposure but may fail to address episodic and mobility-linked contributions that shape total dose.

### Why clean household fuel did not eliminate high-intensity exposure peaks

An important finding was that peak-Qmean exposure did not differ significantly across cooking fuel categories, despite clear differences in median home PM_2.5_ concentrations. This suggests that household fuel type influences routine residential exposure but may be a weaker determinant of short-duration high-intensity exposure events. The persistence of exposure spikes among clean-fuel users indicates that peak exposures are generated by sources operating beyond the immediate household environment.

This interpretation is consistent with our observation that participants experienced higher PM_2.5_ concentrations away from home than within the home. Exposure peaks may therefore reflect interactions with shared community sources, including neighbouring household emissions, market and roadside cooking activities, transport-related pollution, waste burning, and resuspended dust, rather than solely emissions from a participant's primary cooking fuel. In such settings, mobility through multiple microenvironments may diminish the contrast between household fuel groups for peak exposure metrics even when differences remain evident for home-based median exposure.

These findings have important policy implications. While transitions to cleaner household fuels remain essential for reducing chronic household smoke exposure, they may be insufficient to eliminate the episodic exposure events that contribute substantially to personal dose. Effective exposure reduction strategies will therefore require interventions that extend beyond the household and address community-level and mobility-related sources of pollution.

### Limits of ambient proxies and the need for person-centred surveillance

Ambient–personal concordance was modest and meteorology-dependent. Regression slopes rarely approached unity, indicating systematic underestimation of personal exposure by ambient proxies. Wind-sector diagnostics contextualised regional transport influences but also highlighted limited directional variability and the complexity of attributing source sectors using fixed-site monitors alone.

These findings underscore that reliance on large-scale satellite surfaces or sparse ambient networks risks mischaracterising lived exposure. Satellite-derived background concentrations may capture Harmattan dust or regional transport, yet fail to account for indoor cooking, peri-domestic combustion, and mobility-linked microenvironments. Conversely, household-based assumptions overlook background and transport influences.

Person-centred exposure metrics therefore represent not a methodological luxury but a necessary refinement for surveillance, burden estimation, and intervention evaluation in structurally heterogeneous settings.

### Toward a climate-structured and equity-oriented model of personal exposure

Our results both converge with and extend the literature. They align with prior evidence that household energy and poverty shape risk and that dry seasons amplify pollution. However, they depart from the prevailing household-centric lens by:

1. Quantifying the away-from-home contribution to daily dose.
2. Demonstrating the limits of ambient proxies for representing personal exposure.
3. Advancing within-place inequality as a policy-relevant exposure metric rather than a descriptive footnote.
4. Integrating seasonal amplification with structural vulnerability.

By doing so, this study shifts exposure science in Sub-Saharan Africa from static, source-specific frameworks toward a dynamic model integrating climate, mobility, and structural inequity.

### Methodological contributions to exposure assessment in maternal health

This study makes several methodological contributions to exposure science in maternal and perinatal health research, particularly within low-resource and climatically diverse settings.

First, the deployment of minute-resolution wearable monitoring across multiple countries and seasons demonstrates the feasibility and value of high-frequency, person-centred exposure assessment in Sub-Saharan Africa. This approach captures temporal variability and short-duration high-intensity events that are not detectable using conventional ambient monitoring or modelled estimates.

Second, the integration of geolocation data enabled microenvironment classification, distinguishing home and away-from-home exposure contexts. This allows for the explicit modelling of mobility-linked exposure pathways, addressing a critical limitation in exposure assessment frameworks that assume static residential exposure.

Third, the study introduces and applies novel exposure metrics, including the peak-Qmean and the episode-intensity index, which quantify sustained high-exposure periods and the contribution of episodic events to total exposure burden. These metrics provide a more physiologically relevant characterisation of exposure than central tendency measures alone.

Fourth, the analytical framework integrates structural determinants, including wealth, occupation, and rural–urban residence, alongside environmental variability. The use of inequality metrics such as the Gini coefficient and Concentration Index enables quantification of within-population exposure disparities, extending beyond traditional between-group comparisons.

Collectively, these components constitute an adaptable and context-sensitive exposure assessment framework that captures the complex interplay between environmental conditions, behavioural patterns, and structural vulnerability. This framework provides a foundation for improved exposure modelling, epidemiological analyses, and targeted intervention design in maternal and newborn health research.

### Implications for policy and climate-responsive maternal health

Effective mitigation in these contexts must couple clean-fuel transitions with seasonally responsive strategies that target transport corridors, informal markets, and peri-domestic combustion. Interventions should prioritise structurally vulnerable subgroups identified through wealth, occupation, and within-place dispersion metrics.

Embedding scalable personal exposure monitoring into maternal health surveillance systems—supported by local analytic capacity and explicit equity indicators—would yield more accurate burden estimates and more effective, fairer interventions. As climate variability intensifies across the region, seasonally adaptive exposure mitigation will become increasingly important. These may include targeted interventions such as market-level clean energy transitions, traffic emission control during peak mobility periods, and seasonal public health advisories during high-exposure periods such as Harmattan.

### Strengths and limitations

This study benefits from high-resolution personal monitoring across multiple climatic contexts and integration of inequality metrics. Limitations include short monitoring windows per participant, unavailability of ambient data from Mozambique and limited wind-direction variability for inferential analysis. Findings from this study characterise seasonal short-term personal exposure profiles and should not be interpreted as estimates of cumulative exposure across the entire gestational period.

Although GPS-enabled geofencing allowed separation of home and away-from-home exposure periods, the study could not reliably distinguish transport, market, occupational, and other non-residential microenvironments. Consequently, away-from-home findings identify exposure context but not definitive source attribution.

Ambient–personal concordance analyses were limited to The Gambia and Kenya because ambient PM_2.5_ data capture in Mozambique was insufficient for robust paired analysis. This limits cross-country comparability of ambient–personal relationships and prevents assessment of whether the Mozambique personal exposure patterns were reflected in fixed-site ambient measurements. In addition, the sample was not designed to be nationally representative. Rural–urban balance differed by country, and some occupational and fuel-use strata were small. These imbalances may limit generalisability beyond the participating PRECISE/DYAD communities.

Several potentially important behavioural and household-level determinants were not measured with sufficient temporal resolution for direct adjustment, including cooking duration, stove location, ventilation behaviour during cooking, household fuel stacking, second-hand smoke exposure, use of protective behaviours, and detailed time-activity diaries. These factors may confound or modify observed associations between fuel type, microenvironment, and personal PM_2.5_ exposure. Accordingly, analyses are interpreted as descriptive and exposure-characterising rather than causal.

## Conclusion

While clean-fuel transitions in developing countries remain essential to protect the health of susceptible population subgroups, particularly women and young children, they are insufficient without pollution mitigation measures outside of the home, including transport emissions and biomass use for cooking in markets and other public spaces. This may become increasingly important as climate variability alters the frequency, duration, and intensity of high-exposure seasons across many parts of sub-Saharan Africa. Additional targeted action for structurally vulnerable groups identified by wealth, occupation, and place will reduce existing health inequalities. Embedding personal exposure monitoring into national air-quality systems—supported by scalable sensors, local analytic capacity, and explicit equity metrics—would yield more accurate burden estimates and more effective, fairer interventions.

## Data Availability

The datasets generated and analysed during the current study are available upon reasonable request, subject to PRECISE/PRECISE-DYAD data access policies, institutional approvals, and applicable ethical and data governance requirements.
