# Supplementary material for: Unequal exposure: spatio-temporal and structural determinants of personal PM2.5 exposure in three sub-Saharan African geographies—the Gambia, Kenya, and Mozambique
Source: Front Glob Womens Health. 2026 Jul 30;7:1843221. doi: 10.3389/fgwh.2026.1843221 (PMC13469064; doi:10.3389/fgwh.2026.1843221)
Supplement: Supplementary file 1 [file Supplementaryfile1.docx]

**Supplementary Figures**

Supplementary Figure S1: Seasonal contrasts and distribution of personal PM₂.₅exposure

(B) Proportion of person-days exceeding 15 µg/m³ by country and season with 95% Wilson confidence intervals.
(C) Within-participant seasonal differences (Δ = Dry − Wet) in median exposure among participants observed in both seasons. (D) Distribution of participant-day median PM₂.₅concentrations by country and season on a log₁₀ scale, with reference lines at 5 and 15 µg/m³.

Supplementary Figure S2: Concordance between ambient and personal PM₂․₅

*Hourly medians of ambient vs personal PM₂․₅ on log–log axes, stratified by facility and season (panels). Red lines show ordinary least-squares fits with 95% CI bands; equations and adjusted R² are annotated in each panel. Points represent hours with concurrent ambient and personal measurements.*

Supplementary Figure S3: Wind roses for ambient PM₂․₅

Supplementary Figure S4: Fuel-specific exposure and diurnal peak timing across seasons

(A) At-home median PM₂.₅ exposure by primary cooking fuel, stratified by country (log scale; WHO guideline lines shown).
    (B) Season-specific diurnal peak hour of away-from-home exposure, with median peak timing and 95% bootstrap confidence intervals. Colours represent dry and wet seasons.

Figure S5: Stratum-specific inequality in personal PM₂.₅ exposure

**Supplementary Figure S5. Stratum-specific inequality in personal PM₂.₅ exposure.**
Gini coefficients for participant-level daily median PM₂.₅ concentrations are shown across country, rural–urban residence, occupation, and wealth quintile strata. Points represent stratum-specific Gini coefficients and horizontal lines indicate bootstrap 95% confidence intervals. Labels indicate the Gini coefficient and sample size for each stratum. Higher Gini coefficients indicate greater within-stratum inequality in personal PM₂.₅ exposure. PM₂.₅, fine particulate matter.
